# Hydrogeochemical Evaluation of Groundwater Quality Parameters for Ogallala Aquifer in the Southern High Plains Region, USA

**DOI:** 10.3390/ijerph19148453

**Published:** 2022-07-11

**Authors:** Derek Haskell, Joonghyeok Heo, Joonkyu Park, Chao Dong

**Affiliations:** 1 Staff Geologist, Hart & Hickman, Charlotte, NC 28203, USA; derek.haskell25@gmail.com; 2 Department of Geosciences, University of Texas Permian Basin, Odessa, TX 79762, USA; 3 Department of Civil Engineering, Seoil University, 28 Yongmasan-ro-90-gil, Jungnang-gu, Seoul 02192, Korea; 4 Department of Chemistry, University of Texas Permian Basin, Odessa, TX 79762, USA; dong_c@utpb.edu

**Keywords:** Ogallala Aquifer, groundwater quality, statistical analysis, High Plains region, Permian Basin, Texas

## Abstract

The purposes of this study are to analyze the groundwater quality of the Ogallala Aquifer and evaluate the hydrological characteristics in the southern High Plains region of the Permian Basin, Texas. Levels of chloride, fluoride, nitrate, selenium, pH, and total dissolved solids (TDS) were analyzed for the period 1990–2016. Data concerning a total of 133 wells were collected from the Texas Water Development Board (TWDB), which is an open database provided by the US government. The average levels of contaminants were compared to their respective Maximum Contaminant Levels (MCL) stipulated by the Environmental Protection Agency (EPA). The study area experienced high concentrations of most parameters including chloride, fluoride, nitrate, selenium, and TDS, within the contaminants’ respective MCLs. Borden and Dawson counties experienced the highest overall amounts of groundwater pollutants. Possible sources of each contaminant are discussed, with oil and gas activities, agricultural practices, and other human actions impacting the conditions. This research provides important information about groundwater quality of the Ogallala Aquifer and contributes to understanding the response to development in the Permian Basin, Texas.

## 1. Introduction

Water is the most vital natural resource present on Earth. The Ogallala Aquifer (also known as the High Plains Aquifer) is one of the largest aquifers in the world. The aquifer extends from South Dakota through Texas across portions of eight states in USA, so it is crucial that it is kept sustainable. With the presence of oilfield activity, the Ogallala Aquifer is increasingly susceptible to contamination. Irrigation and the use of fertilizers is very common in the High Plains. The water quality of the Ogallala Aquifer is often sufficient for irrigation purposes, but in most places it does not meet drinking water standards set by the U.S. Environmental Protection Agency (EPA) for parameters like chloride, fluoride, and total dissolved solids (TDS) [1].

The Ogallala is an unconfined aquifer that has seen drastic changes in its properties throughout the years, due to agricultural processes and other activities [2]. Hornbeck and Keskin [3] described how center pivot irrigation technology greatly improved through the 1950s. This new technology was manufactured and distributed on a large scale, which led to a five-fold increase in groundwater withdrawal rate from 1949 to 1974. The US Geological Survey (USGS) [4,5] conducted a study that measured water levels from the 1950s to 2015, based on 3164 wells, and showed that the Ogallala Aquifer demonstrated an average 234 feet decline in water level across Texas. Based on data from 7524 wells from 2013 to 2015, there was a decline in the water level with an average of 33 feet across Texas. In 2015, compared to predevelopment levels, the saturated thickness across Texas declined from 10 feet to more than 50 feet [4,5]. The declines in water level, saturated thickness, and recoverable water throughout Texas are very alarming, and action to combat this is imperative. 

The Permian Basin in Texas is well known throughout the world for producing large quantities of oil and gas. Exploration for oil in the Permian Basin has been occurring for approximately a century. Unconventional drilling methods including horizontal drilling, hydraulic fracturing, and other advanced technologies, have greatly increased oil production in the past decade [6]. Hydraulic fracturing alone has had a 10-fold increase from 2000 to 2015 in the United States [7]. The population of the Permian Basin is continuously growing, with a total of 2,061,422 residents in 2018 [8]. From 2013 to 2018, the population grew 3.3%, which added 65,942 residents, and another 2.3% increase is expected by 2023. 

There are many environmental concerns relating to the Permian Basin, Texas, especially when oil and gas activity is so prevalent [9,10]. Also, the continuous increase in population and agriculture are causes for concern [11]. Some contaminants can be found naturally in bodies of water, but the biggest causes of contamination are anthropogenic, including agriculture, littering, and industrialization [10]. Epstein [9] mentioned that many reserve pits are known to leak, and groundwater and sources of drinking water are contaminated with pollutants such as arsenic, benzene, chromium, lead, and other metals. These pits can overflow due to heavy rain and flooding [11,12,13]. The failure of casing and cementing technologies, although a very low possibility, can be a further cause of groundwater contamination. Old pipes in the public water system can corrode over time, leading to an increase in contaminants such as copper and lead. Agriculture is one of the major industries in the Southern High Plains region, and plays a pivotal role in the economy. Robertson and Sharp [14] conducted a study highlighting how synthetic and natural (manure-based) fertilizers, specifically nitrates, were the primary anthropogenic culprits for groundwater contamination. Synthetic fertilizers have been commonly used since the 1950s, becoming the primary choice of fertilizer. Total commercial fertilizer use greatly increased until approximately the 1980s, and has since fluctuated due to supply and demand [15].

In the past decade, hydraulic fracturing has become a beneficial technique for extracting oil and gas from reservoir rocks that have very low permeability and porosity [16,17,18]. Hydraulic fracturing is the process of creating fractures by injecting fluids, mostly composed of water, sand, and chemicals, into reservoir rocks that lack porosity and permeability [16]. Jasechko and Perrone [7] conducted a study that demonstrated how hydraulic fracturing wells in a proximity of approximately 1–3 km of domestic groundwater wells can impact groundwater contamination. It was also shown that conventional oil and gas wells were in a proximity to water wells and posed a risk of contaminating groundwater. Oil refineries, like the one in Big Spring, Howard County, Texas for example, can also prove hazardous not only to water quality, but also to air and soil. These refineries can be a substantial cause of the release of toxic compounds such as BTEX (benzene, toluene, ethylbenzene, and xylene) [19]. Water discharge from these refineries is strictly regulated by the Safe Water Drinking Act (SWDA) and Clean Water Act (CWA); however, contamination from previous discharge can remain in water bodies [19,20]. 

There have been numerous research studies into groundwater qualities and contaminations in Texas [2,3,9,10,11,12,13]. Some have been undertaken to evaluate the groundwater quality for Ogallala Aquifer in the Southern High Plains region after anthropogenic influences such as oil production or agriculture [16,17,18]. However, few studies have monitored groundwater quality parameters in West Texas related to the High Plains regions. Moreover, previous research has tended to evaluate a single parameter in the observation of long-term change. Therefore, human interventions or natural variations (i.e., multiple parameters) affecting the Ogallala Aquifer in the Southern High Plains region were not appropriately considered in previous research. With these concerns and facts stated, the objective of this study was to assess the hydrogeochemical characteristics and groundwater quality of the Ogallala Aquifer in the Southern High Plain region of Texas. Since the petroleum and agriculture industries are such a vital portion of the economy in this region, and groundwater resources are so crucial, it is important to determine the quality of groundwater here. A long period of groundwater data with a relatively dense observation network is available for the study area. Because of its hydrological characteristics and the availability of quality groundwater data, the study area was used to evaluate the impact of various phenomena including human activities and natural factors on groundwater quality in the Ogallala Aquifer in the High Plains Region, USA. The contaminants chloride, fluoride, nitrate, and TDS, along with pH, were analyzed from approximately 1990 to 2016 to determine whether groundwater was safe to consume for the populations of the respective counties. With this data, an evaluation was made of the impact of energy development and agricultural practices, to determine the effect on groundwater resources in the study area. This research provides important information for groundwater resource management and contributes to understanding groundwater responses to energy and agricultural development in the Permian Basin and Southern High Plains.

## 2. Study Area

**Figure 1:**
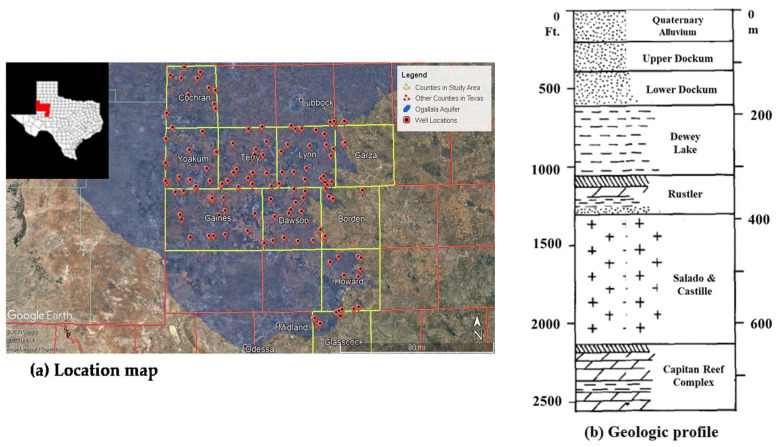
(**a**) Location of groundwater wells and (**b**) the geologic profile in the study area including the underlying Ogallala Aquifer (cited from Shannon et al. [2] and Nelson et al. [11]).

**Figure 2:**
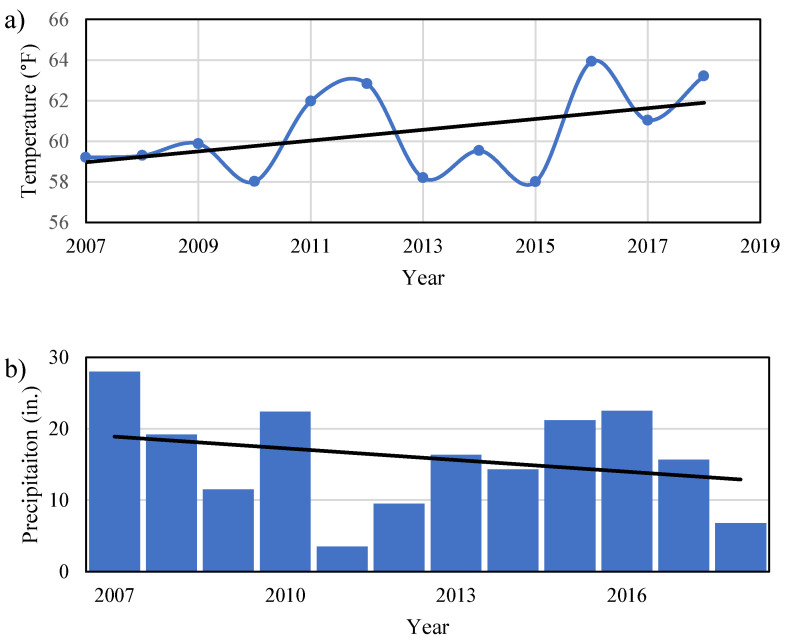
(**a**) Annual average temperature (trend equation: y = 0.27x − 474.57) and (**b**) annual total precipitation (trend equation: y = −0.55x + 1112.80) in Lamesa, TX according to US Climate Data from 2007 to 2018.

The study area consisted of the southern-most portion of the High Plains, located in the Permian Basin, in West Texas, USA. Ten counties comprised the study area: Borden, Cochran, Dawson, Gaines, Garza, Glasscock, Howard, Lynn, Terry, and Yoakum (Figure 1a). These counties combined have an area of 9350.1 square miles. Most of the study area is a rural environment and is slightly populated. The population of the study area has generally increased, as it has for the whole of the Permian Basin in Texas, by a range of 0.6% to 22.6% from 2010 to 2019 [21,22]. The Ogallala Aquifer provides more water for the state of Texas than any other aquifer. Precipitation is uncommon, with an average rainfall for all 10 counties ranging from around 18–21 inches per year [23]. In the eastern side of the study area, there is a large increase in closed drainage depressions compared to the western side, which can fill with water after precipitation [24,25]. The year 2007 had the greatest amount of precipitation with ~28 inches of rain, while the highest average temperature of ~64 °F occurred in 2016. Most of the precipitation occurred between the months of May and June, while March, July, and September contributed to a lesser degree (Figure 2).

Figure 1b illustrates a geological structure in the study area. Geologically, the study area is situated on the western side of the Monument Draw trough, on the shelf margin between the Central Basin Platform and the Delaware Basin, on the western side of the Permian Basin. This region has a complex and varied geological history; deposition began during the Paleozoic Era, in a stable and shallow marine environment. The Ogallala is an alluvial aquifer consisting of sand, silt, and gravel. It is an unconfined aquifer characterized by unconsolidated alluvial and eolian sediments consisting of interbedded sand, silt, clay, caliche, and gravels. Average hydraulic conductivity was calculated to be 2.6 m per day, with estimated transmissivity values of less than 1.0 to greater than 4267 m per day [2,11]. The formation was deposited during the late Miocene to early Pliocene and extends for approximately 450,000 km^2^. The aquifer was formed during the early to middle Cretaceous, with the main water bearing units being dolomite, limestone, and sands [10,11]. 

As a result of interactions with the atmosphere, the surficial environment, soil, and bedrock, a wide range of different chemicals can be dissolved in groundwater. Groundwater tends to have much higher concentrations of most constituents than surface waters do, and deep groundwater that has been in contact with rock for a long time tends to have higher concentrations of constituents than shallow water. The Ogallala Formation has a thickness from 0 to approximately 800 feet, with an average saturated thickness of 95 feet [26]. Throughout most of the Ogallala Aquifer area, withdrawal of water has exceeded the recharge rate. Water levels have declined in excess of 300 feet in the last 50 to 60 years. The use of groundwater from the Ogallala is mainly dominated by agriculture, with ~95% of the groundwater used for agricultural purposes. The remaining ~5% of groundwater use includes livestock production, oil and gas production, manufacturing, and wholesale and retail trade.

## 3. Data and Methods

All groundwater data was acquired from the Texas Water Development Board (TWDB) Groundwater Database (GWDB) which is an open database provided by the US government. We collected all historically available data for our study area from TWDB-GWDB, and checked the location of their wells. Most data for pollutants were consistently provided every four years starting at 1990. All ten counties had data for their pollutants in 2016, except for Borden, Glasscock, and Howard, which had their latest data recorded in 2012. For this reason, we finalized the total 133 wells to evaluate the groundwater quality and analyze hydrological characteristics throughout the study area. 

The database of water quality by county can display information for every county in Texas, the aquifers that underlie each county, and the coordinates of the water wells that have been drilled and tested in each county. All parameters can be acquired from this database and can be filtered out manually if data for certain contaminants are not required. This database also provides state well numbers, aquifer codes, parameter codes, reliability, well depths, dates of wells tested, and other information. The data collected in this study was transferred to Excel and rearranged, for easier utilization and interpretation. The years selected in this study were based on data availability, but some data for periods in between was also available. Average depths of these wells were calculated for each county, along with the contaminant parameters: chloride, fluoride, nitrate, selenium, pH, and TDS. These contaminants were analyzed to determine the average values for each parameter for the respective year in each county. 

The averages of each parameter were further assessed where changes in groundwater levels throughout time were made evident, compared to their respective maximum contaminant level (MCL). The MCL was established by the EPA within the Safe Water Drinking Act (SWDA), which was passed in 1974. With the line graphs, interpretations were made to indicate which counties had exceeded their MCL for each parameter and which counties did not. Every parameter was then displayed for each county, and a range of values were visualized, making it possible to see the maximum and minimum values, the 25th and 75th percentiles, and the medians. This data provided the statistical values of groundwater quality for each county. This included the actual minimum and maximum values of each parameter for each year (not just the averages), the standard deviation, and range. Standard deviation was calculated using the sample standard deviation method, because not every well in each county was analyzed.

## 4. Results and Discussion

### 4.1. Chloride 

**Figure 3:**
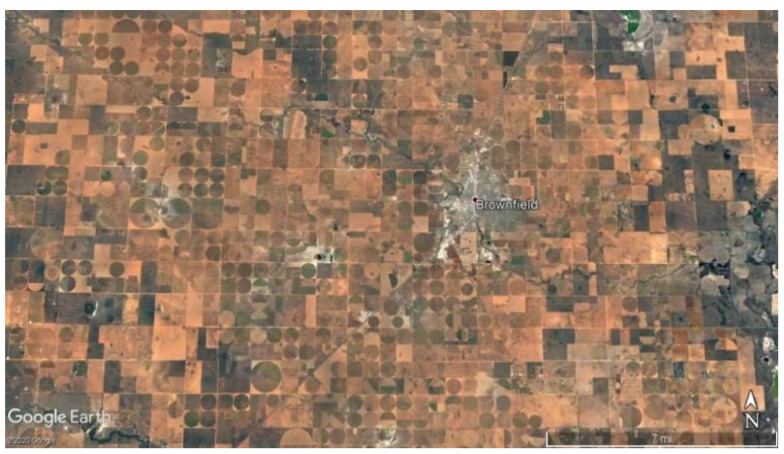
Crop circles in Terry County, Texas (image from Google Earth).

Chloride is a naturally and anthropogenically occurring anion that is mostly known for being combined with sodium to form halite or salt. Chloride is commonly found in oilfield brines and evaporites, which are common constituents in the Permian formations underlying parts of the Ogallala [27,28,29]. This anion can often be found in marine shales from seawater, where chloride gets trapped during deposition [27]. In semi-arid regions such as the study area, the soils are not fully leached, which allows an accumulation of solutes. Then, when the land gets irrigated, the rates of leaching increase and in turn increase the buildup of solutes such as chloride in groundwater. There are many agricultural practices in the study area that support this claim, with an example from Terry County showing an overview of the substantial amount of crop circles where irrigation occurs (Figure 3). Freeman [30] went on to confirm that halite contamination in shallow groundwater systems is impacted by road salt, and by the dissolution of natural halite invading aquifers, and groundwater is affected by discharge from water softening systems in houses. Road salt or rock salt is salt in its natural form, made of sodium chloride. It can be found all over the USA, including Texas. The formation process happens in sedimentary mineral beds when lakes or seas dry up. There are deposits ringing dry lake beds, inland marginal seas, and enclosed bays and estuaries in arid regions, where the salt crystallizes out of evaporating brine lakes.

The MCL for chloride is 250 mg/L [20]. This elevated level of chloride tends to cause a salty taste in drinking water. If chloride concentrations reach 350 mg/L, the groundwater becomes unacceptable for industrial use as high concentrations can cause corrosion in pipes. With these levels of concentrations in mind, oilfield brines typically consist of chloride concentrations of 50,000 mg/L [28]. Chloride can be combined with other ions to form compounds used in products such as salt, dry-cleaning agents, plastics, dyes, and synthetic rubbers [30,31]. 

**Figure 4:**
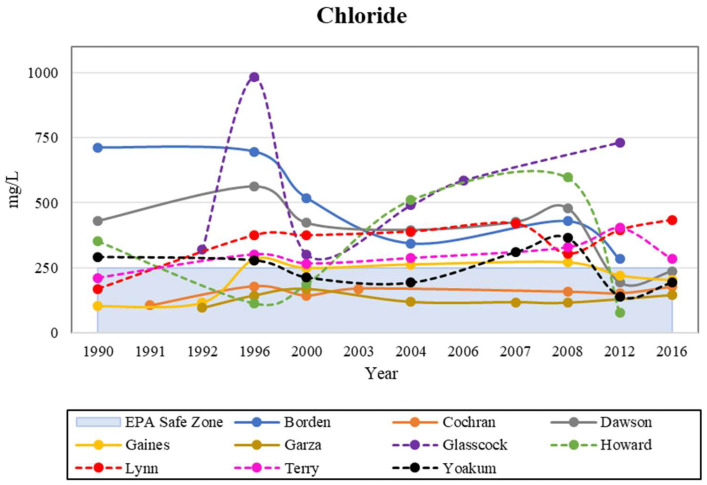
Historical analysis of chloride levels in the study area, compared with the EPA safe zone.

In this study, only the counties of Cochran, Dawson, Gaines, Garza, Howard, and Yoakum fell within the MCL safe zone for chloride in 2012 and 2016 (Figure 4). The study showed chloride concentrations in the Southern High Plains. Many of the chloride concentrations were below the MCL of 250 mg/L, but there was a concentration of approximately 10 times that value. The Northern High Plains (the states of North Dakota, Nebraska, and Colorado) was less susceptible to high concentrations of chloride, while the Southern High Plains (Texas, Oklahoma, and New Mexico) experienced more concentrations exceeding the MCL [28,29]. There is considerable crop cultivation near the study area, revealing an overview of crop circles where irrigation occurs [29]. Our study area had a higher level of chloride than other areas, because Borden, Dawson, and Glasscock Counties have relatively high levels of human intervention, including agriculture, oil and gas production. Freeman [30] went on to confirm that halite contamination in shallow groundwater systems is impacted by road salt, and by the dissolution of natural halite invading aquifers. A study by Reedy et al. [32] showed concentrations of chloride with a median of 31.3 mg/L, and a maximum value reaching 13,898 mg/L. These high values in the Southern High Plains could be a result of wells being at shallower depths. Influence from underlying formations, an upward hydraulic gradient that is intensified by pumping, and oilfield activity can lead to high amounts of chloride contamination in the Ogallala within the study area. 

### 4.2. Fluoride

Fluoride is an inorganic, naturally and anthropogenically occurring ion that is found along the natural surface and in groundwater [33,34]. Fluoride can originate from the dissolution of fluorite, fluoropatite, and certain silicates. Mukherjee and Singh [35] confirmed this path of fluoride contamination, as the dissolution of fluorapatite by Pseudomonas fluorescens contributes to fluoride in the environment. Fluoride can have beneficial effects when taken in moderation, such as improving dental health and bone mineralization [29,33]. Drinking water is the primary pathway for intake and exposure to elevated levels of fluoride [34]. 

**Figure 5:**
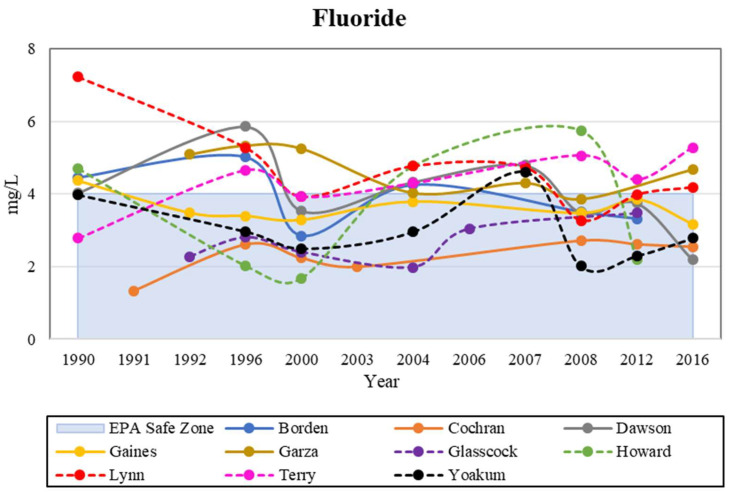
Historical analysis of fluoride levels in the study area, compared with the EPA safe zone.

The measurements for fluoride in our study area generally exceeded their MCL. Previous studies showed an increase in concentrations in fluoride in the Southern High Plains [28,32,36,37]. The average median value of fluoride was 2.4 mg/L, while the maximum value was 9.8 mg/L, more than twice its MCL. Hudak [28] observed a maximum value of fluoride resulting in levels approximately four times greater its MCL in the semi-arid environment of the High Plains region. According to the data compiled in this study, fluoride’s MCL was only exceeded in the counties of Garza, Lynn, and Terry (Figure 5). While fluoride concentrations have remained relatively consistent throughout the study area, Lynn, Howard, and Terry Counties accounted for the highest accumulation of fluorides. Concentration limits remained relatively consistent from 1990 to 2008, well above the MCL of 4.0 mg/L. This could result in high concentrations of fluoride in shallow groundwater environments if these conditions are present [11,16]. Fluoride mobility from soil to water is impacted by adsorption and leaching processes, which thrive in a semi-arid to arid environment with alkaline soils [11,16]. 

### 4.3. Nitrate

Nitrate is an inorganic, naturally and anthropogenically occurring compound that is common throughout the world and vital for life [20]. Nitrate is used extensively in fertilizers, which seem to be the main source of nitrate contamination throughout the world. This compound moves very easily after entering groundwater, as it neither absorbs nor precipitates on solids in the aquifer [38,39]. If crops are grown in a soil enriched with fertilizers, a mechanism is created for nitrates to infiltrate the groundwater. Prime examples of a potential mechanism are center pivot irrigation systems, which move in circles spraying water on crops. 

**Figure 6:**
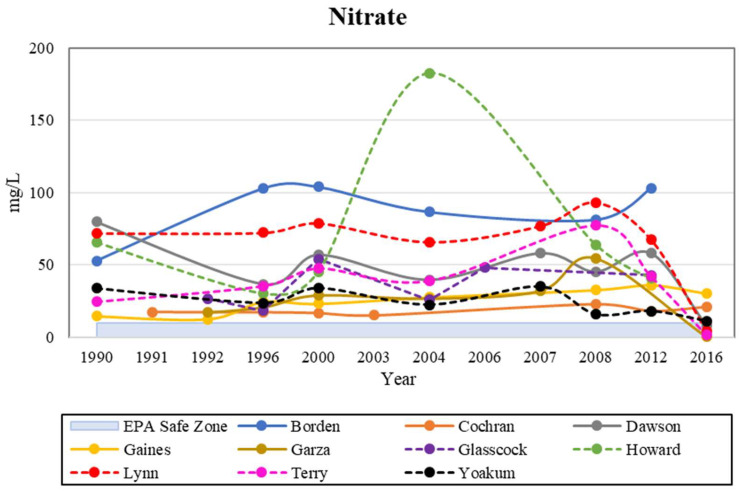
Historical analysis of nitrate levels in the study area, with the EPA safe zone shown for comparison.

Texas is ranked in the top five states in the US for total crop exports, with over 194,000 farms covering approximately 77% of the land in the 2000s [22,28]. There are six possible sources of nitrate in the arid environments of west Texas: (1) sewage waste from septic tanks or fields, (2) atmospheric deposition of reactive nitrogen, (3) movement of mineralized nitrogen, (4) microbial fixation, (5) accumulation of nitrate in desert soils, and (6) anthropogenic fertilizers. Among these potential sources of nitrate contamination, anthropogenic fertilizers are the main culprit due to their widespread use and seasonal repetition [14]. The possibility of sewage waste being the main source of nitrate contamination can be ruled out, because heavily populated areas, such as Dallas and Houston, experience the most sewage waste yet have no significant groundwater–nitrate problems when compared to the study area. In this study, every county consistently exceeded the MCL for nitrate, except in 2016 where Dawson, Garza, Lynn, and Terry Counties fell below the MCL (Figure 6). It is important to note that almost all counties began a downward trend after 2008, with the exception of Borden County. Howard County experienced a very high spike in 2004, which could be due to an increase in precipitation that year as the average water level was closer to the surface than in the years before and after 2004. Our study area is predominantly arid or semi-arid. The arid climate usually receives less than 10 inches (~250 mm) of precipitation in an entire year. In 2004, the annual total precipitation for the study area was 27 inches (~685 mm) [18]. When compared to average years, 2004 had more than double the amount of precipitation. Due to an increase in precipitation, pollutants on the surface display a stronger ground-infiltration mechanism. 

Total nitrogen is a frequently assessed component of sediments and soils that can be used to distinguish sources of organic matter, environmental depositional conditions, pollution indices, and soil quality indicators. It transports and infiltrates into ground with precipitation. In 2004, the levels of nitrate reached about 170 mg/L (=ppm). The high concentrations of nitrates in 2004 could have been from agricultural runoff from either animal waste or inorganic fertilizers (Figure 6). However, those concentrations could have also been from weathering, which can increase nitrate levels in water systems. If ingested at high levels, the nitrates will turn into nitrites, which are a more toxic form that could result in brain damage and other long-term effects [15,18]. 

### 4.4. pH

**Figure 7:**
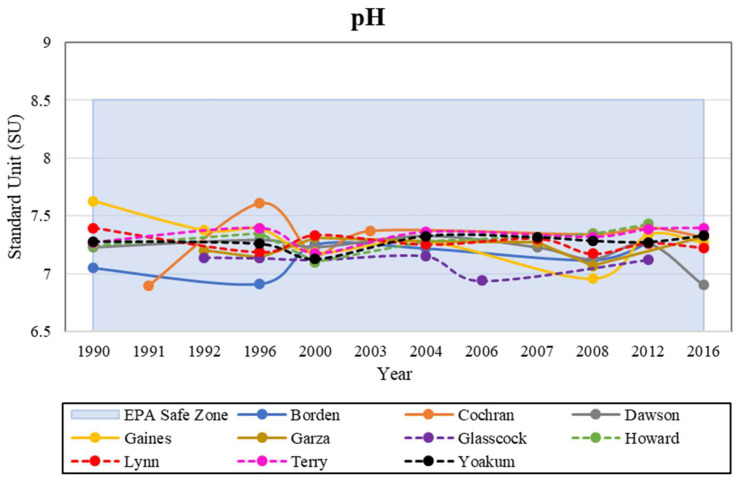
Historical analysis of pH levels in the study area, within the EPA safe zone.

pH is a measure of how acidic or basic a water solution is. It is measured on a scale that ranges from 0 to 14, with 0 being most acidic, 7 being neutral, and 14 being most basic, and measures how many hydrogen or hydroxide ions are in water [40]. Appleyard et al. [41] described how the acidity of soil and groundwater can increase if an aquifer is of sandy composition with no carbonate content to serve as an acid buffer. This same study showed that soils less than 2 m from the surface experienced little pH change, which could be due to organic matter and biological activity. When depths became greater than 2 m, pH levels started to become more acidic. Based on the data, concerns about pH quality in our study area were nonexistent as all counties fell within the pH legal limit of 6.5 to 8.5 standard units (SU) set by the EPA (Figure 7).

### 4.5. Selenium

Selenium is a naturally occurring substance found in the Earth’s crust that is essential for life. It can be anthropogenically produced, as it can form as a byproduct from the mining of sulfide ores. Arid environments that typically see high concentrations of gypsum and soluble salts such as magnesium sulfate and thenardite, allow easy access for selenate to fuse with these minerals. Mills et al. [42] stated that an increase of nitrate can lead to an increase in mobility of selenium in shallow groundwater aquifers, like the Ogallala. 

**Figure 8:**
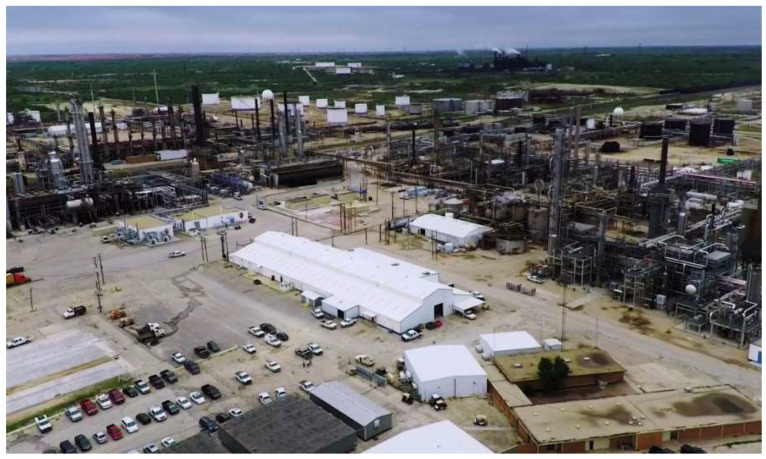
Oil Refinery in Howard County, Texas (image from Google).

Potential sources of inflated selenium concentrations include the leaching of marine shales, irrigation water and fertilizers, and discharge from oil refineries and mines (Figure 8). Soluble salts were found to be the dominant source of selenium contamination, and the selenium in gypsum had very little impact on contributing to the concentrations in groundwater. There is a weak relationship between selenium concentrations on the surface or in the unsaturated zone and selenium in groundwater. Organic matter is a major factor in high selenium concentrations and distribution [43,44]. 

**Figure 9:**
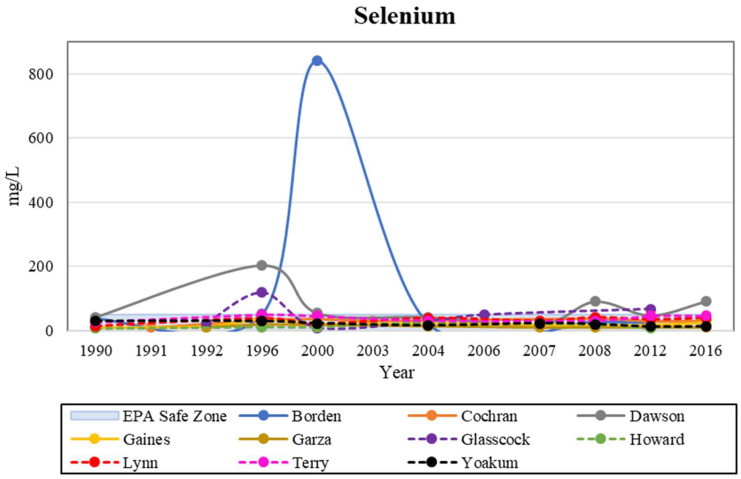
Historical analysis of Selenium levels in the study area, with the EPA safe zone shown for comparison.

The most common symptoms of selenium toxicity observed were diarrhea, fatigue, hair loss, joint pain, nail discoloration or brittleness, nausea, and headaches [45,46]. Previous research reported generally higher selenium concentrations in the Southern High Plains [11,16,37]. Our study also indicated that the greatest selenium concentration was approximately five times higher than its MCL. Figure 9 represents selenium levels; during the observation period, all counties succeeded in staying below the MCL of 50 mg/L, except for Borden, Dawson, and Glasscock. This increase suggests that the construction of oil refineries had an influence on selenium contamination in the study area. For example, oil refineries such as the one located in Howard County (Figure 8) in our study area can be potential sources for selenium contamination [43]. Although the refineries are under strict regulations from the SWDA and CWA when it comes to how they discharge water, off-site contamination such as accidental leakage can occur [19].

### 4.6. Total Dissolved Solid (TDS)

**Figure 10:**
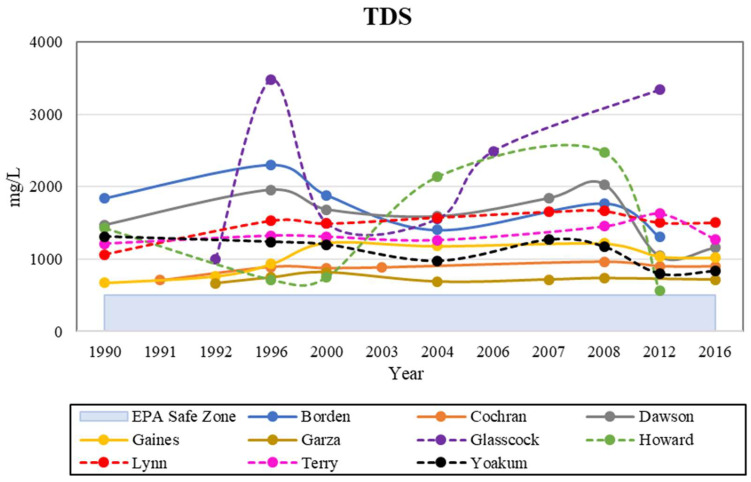
Historical analysis of TDS levels in the study area, with the EPA safe zone indicated.

TDS is not only an indicator of salinity, but is a measurement of all the dissolved inorganic and organic substances in water [47]. Potential sources of elevated TDS concentrations in the study area include mineral fertilizers and underlying bedrock units, such as the Dockum Aquifer and Permian evaporates [48]. The Dockum Aquifer has TDS concentrations ranging from 1–35,000 mg/L in the study area [25]. Other possible sources of salinity include brine pits from old oil and gas wells, and the upward mobilization of oil and gas from wells that were not properly plugged [49]. The increase in solutes due to evaporation and the occasional flushing of salts by irrigation water is one of the primary mechanisms of elevated salt concentrations in shallow groundwater [11,48,49]. In this respect, the Southern High Plains seems to be of greater concern than the Northern High Plains, as 84% of observations exceeded the brackish water limit of 10,000 mg/L. A saline plume exists along the northeastern margin of the Southern High Plains which could result in elevated concentrations around the plume [49]. We also observed that the groundwater quality diminished in the shallow observations compared to deeper observations, which gives reason to believe that the source of contamination is primarily at the surface rather than deep in the subsurface. A MCL of 500 mg/L was set for TDS and all counties were consistently above that limit (Figure 10).

### 4.7. Groundwater Quality Parameters and Depth Correlations

**Figure 11:**
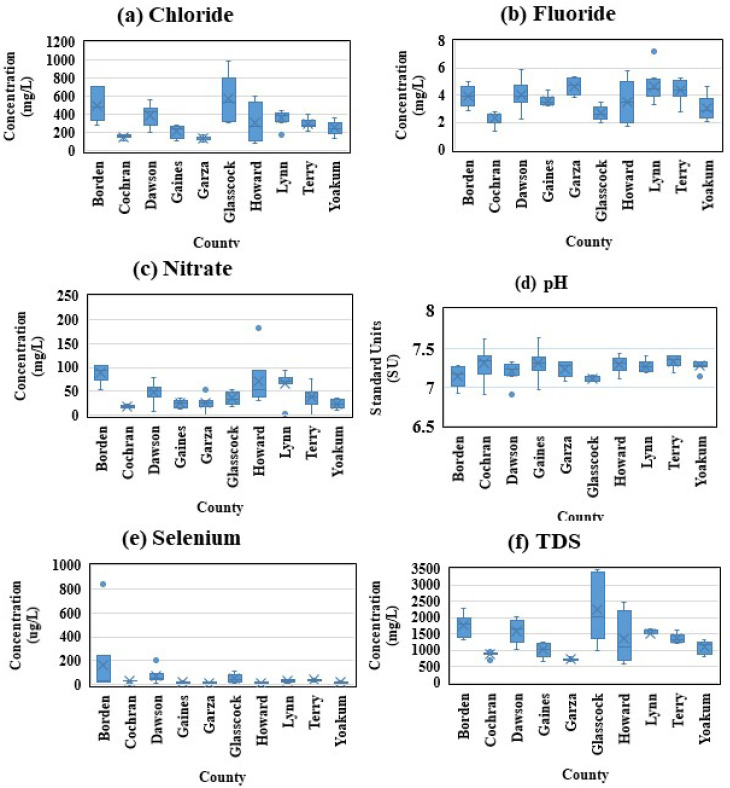
Box and whisker plots of (**a**) chloride, (**b**) fluoride, (**c**) nitrate, (**d**) pH, (**e**) selenium, and (**f**) TDS parameters recorded for each respective county during the research period 1990–2016.

Box and whisker plots were compiled together, more efficiently highlighting each pollutant and the spread of the values for all counties in this study. The data were used to form these box and whisker plots, which show the minimum and maximum values, the 25th and 75th percentiles, and median. The box and whisker plots allow more efficient visualization of which counties had greater values of pollution than others (Figure 11). Statistical mean values were calculated for each parameter in the respective counties to further reinforce the understanding of the data (Table 1). 

Associated oilfield facilities grew, accounting for the increase in production in Texas. These factors resulted in a considerable change in both economic growth and land cover from the 1990s to the 2010s, as the amount of developed land in the Permian Basin area of Texas increased by 11.78 km^2^ or 176% [11]. The oil shale boom resulted in a noticeable increase in well sites and production pads scattered throughout west Texas, contributing to the growth of developed land. The disposal of oilfield brine resulted in higher concentrations of TDS in northwest Texas [2,9]. Fluids associated with the production of oil and gas commonly include sodium chloride, in saline and brine. 

With growing demand from a rising population, the percentage of crop production area increased by 14% from 1992 to 2011 (from 38.5 to 43.9 km^2^), primarily within Martin County [11]. Within this arid region, the primary water source is the Ogallala Aquifer, which provides the necessary groundwater vital for the production of various crops [2,9,11]. The development of center pivot irrigation in the 1950s supported farmers’ endeavours to establish crops in these arid regions. Increased agricultural activity can result in higher concentrations of nitrates and total dissolved solids (TDS).

**Figure 12:**
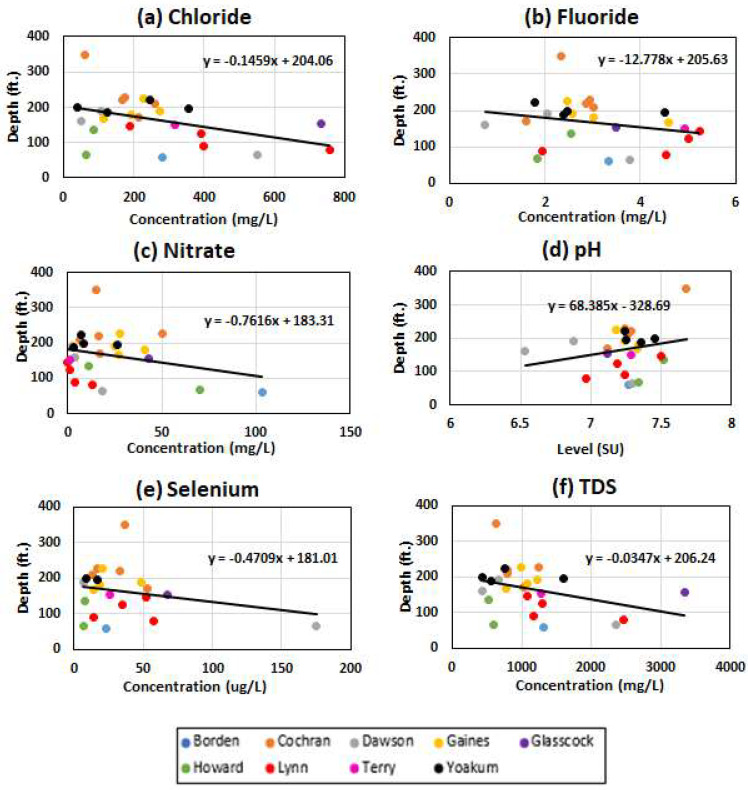
The latest data available for (**a**) chloride, (**b**) fluoride, (**c**) nitrate, (**d**) pH, (**e**) selenium, and (**f**) TDS values versus well depth for each county excluding Garza County during the research period 1990–2016.

In order to further understand the relationship between depth and groundwater quality, correlations between these two parameters were graphed to determine if the source of contamination was at the surface or within underlying formations (Figure 12). The groundwater data for each county with the latest figures for 2012 were used, because these were the most recent data available. Garza County could not be included in this portion of the study due to lack of information on the known depth of the wells. A linear regression line was calculated and graphed to determine whether the data in this study would follow suit. The data collected in our study area showed a decreasing linear regression for all parameters excluding pH, which suggests that the contamination comes from the surface rather than the underlying formations. Groundwater contamination is typically worse in shallower wells compared to deeper ones, due to human impact that occurs at the surface [11,16]. Consequently, deep aquifers should present lower levels of pollution; this relationship shows that deep aquifers are expected to present lower levels of contamination. The pH in the study area ranged from 6.7–8.1 SU (Figure 7), and the deeper wells were more alkaline than the shallower wells. This increase was a result of the aquifer’s composition, with dissolving limestone and dolomite minerals contributing to its alkalinity. In arid climates, the low precipitation results in soils that are closer to neutral or slightly alkaline are due to the weathering and leaching effects of precipitation. The weathering of parent material results in the formation of soil horizons and is a contributing factor to the soil’s pH [11].

A wide range of different chemicals can be dissolved in groundwater as a result of interactions with the atmosphere, the surficial environment, soil, and bedrock. Groundwater tends to have much higher concentrations of most constituents than surface waters do, and deep groundwater that has been in contact with rock for a long time tends to have higher concentrations of the constituents than shallow water does. Shallow groundwater consists of Ca (calcium)–Na (sodium)–HCO_3_ (bicarbonate) dominantly formed by the interaction of atmospherically recharged meteoric water with the soil and shallow bedrock. These waters are usually fresh, but upwelling of deeper saline fluids or saline intrusions from adjacent seawater bodies can influence their chemical composition [11]. Intermediate or deep groundwater rapidly increases in concentration of constituents, primarily by the addition of SO_4_ (sulfate) and Cl (chloride).

## 5. Conclusions

This study analyzed the groundwater quality of the Ogallala Aquifer in the Southern High Plain region of the Permian Basin, Texas from 1990 to 2016. Increase in temperature and a decrease in precipitation indicate a shift towards a more arid climate (Figure 2). This analysis confirms a shift to a more arid climate within the study area. Land cover is predominantly grass and shrub, followed by barren land, then developed land and crops. This area is characterized by high volumes of oil and gas exploration, drilling, and production activities. The semi-transient population varies in conjunction with oil and gas operations. The study area experienced high concentrations of groundwater contamination for most parameters (chloride, fluoride, nitrate, selenium, TDS), except for pH for which all counties were within the contaminants’ respective MCL. According to the standards set by the EPA, the groundwater in most counties would be considered unsafe to drink without some sort of filtration (such as reverse osmosis, anion exchange, lime softening [20]), according to analysis of the final year of data for each county. Regarding the main culprits behind the high levels of groundwater contamination, human activities are either moderately or mostly to blame. This does not rule out the fact that there are some naturally occurring reasons for elevated levels of groundwater contamination. Agricultural fertilizers and sewage waste from septic tanks are significant contributors of contaminants, due to their wide-spread use and seasonal repetition. In addition, natural events like precipitation can be contributing factors for groundwater transformation.

Pollutants such as arsenic, chloride, fluoride, hardness, selenium, and TDS took a downward trend in the later years of the study period. Nitrate and pH levels stayed relatively the same and no trend was observed. In 2012, Glasscock County experienced its highest levels of pollutants including fluoride, hardness, and lithium, and its second highest for chloride and TDS. Arsenic, nitrate, and pH in Glasscock County did not experience any significant trends. Selenium had its peak in 1996, followed by a sharp decline in 2000, but was an increasing trend towards 2012, the most recent year included in the study. In 2012, Howard County observed its lowest levels of arsenic, chloride, hardness, and TDS, and its second lowest levels of nitrate and selenium. In Howard, Lynn, and Terry Counties, no significant trends were observed for fluoride or pH, but lithium observed a sharp decline from 2008 to 2012. In Yoakum County, arsenic, hardness, nitrates, selenium, and TDS underwent decreasing trends from 1990 to 2016. Fluoride and pH showed relatively no change, while chloride and lithium experienced no trends.

Statistical methods were proven to be helpful for analyzing datasets with information of different sizes. Borden and Dawson Counties experienced the overall highest amounts of groundwater pollutants. Pollutants such as chloride, fluoride, and TDS trended downward in more recent years, and natural events like precipitation may be a contributing factor in this progression. The significance of this research highlights the elevated concentrations of groundwater contamination in the Southern High Plains region. Historical data for each county presents trends of groundwater contamination, and possible reasons for some years having exhibited higher concentrations than others. This study evaluated the changes in groundwater quality parameters and identified the impact of human interference and natural factors on groundwater in the study area. Thereby, this research provides important information for groundwater quality management in the Southern High Plains region, and contributes on understanding of the responses of groundwater resources in the Permian Basin, Texas.

## Figures and Tables

**Table 1:** Statistical average values of groundwater quality parameters in all counties in the study area during the research period 1990–2016.

	Borden	Cochran	Dawson	Gaines	Garza	Glasscock	Howard	Lynn	Terry	Yoakum
Chloride (mg/L)	497.7	153.8	393.6	213.4	128.4	568.8	305.5	357.5	297.3	247.5
Fluoride (mg/L)	3.9	2.3	4.0	3.6	4.7	2.7	3.5	4.7	4.4	3.0
Nitrate (mg/L)	88.4	18.3	48.0	25.2	25.9	36.0	71.5	66.4	38.2	24.3
pH (SU)	7.2	7.3	7.2	7.3	7.2	7.1	7.3	7.3	7.3	7.3
Selenium (µg/L)	167.0	29.8	73.7	20.5	13.7	49.6	13.6	32.6	39.9	20.9
TDS (mg/L)	1749.7	873.2	1597.0	1006.1	725.9	2228.0	1346.4	1497.1	1351.8	1099.1

## Data Availability

Not applicable.
